# Safety and accuracy of transbronchial lung cryobiopsy in diagnosing desquamative interstitial pneumonia

**DOI:** 10.1111/crj.13483

**Published:** 2022-03-01

**Authors:** Jun‐ge Zhao, Guo‐wu Zhou, Ling Zhao, Min Liu, Yan‐hong Ren, Hua‐ping Dai

**Affiliations:** ^1^ Department of Pulmonary and Critical Care Medicine, Center of Respiratory Medicine, China‐Japan Friendship Hospital; National Clinical Research Center for Respiratory Diseases, Institute of Respiratory Medicine Chinese Academy of Medical Sciences Beijing China; ^2^ Department of Pulmonary and Critical Care Medicine Zheng‐Zhou Central Hospital Affiliated to Zhengzhou University Zhengzhou China; ^3^ Department of Pathology, Center of Respiratory Medicine, China‐Japan Friendship Hospital; National Clinical Research Center for Respiratory Diseases, Institute of Respiratory Medicine Chinese Academy of Medical Sciences Beijing China; ^4^ Department of Radiology, Center of Respiratory Medicine, China‐Japan Friendship Hospital; National Clinical Research Center for Respiratory Diseases, Institute of Respiratory Medicine Chinese Academy of Medical Sciences Beijing China

**Keywords:** Desquamative interstitial pneumonia, diagnostic accuracy, multidisciplinary discussion, surgical lung biopsy, Transbronchial lung cryobiopsy

## Abstract

**Introduction:**

Transbronchial lung cryobiopsy (TBLC) is a new technique to obtain specimens for diagnosis of interstitial lung disease (ILD) in recent years. The objective of this study is to evaluate the safety and the diagnostic accuracy of TBLC in patients of desquamative interstitial pneumonia (DIP).

**Methods:**

In this study twelve patients confirmed with DIP were selected from January 2019 to December 2020 at the department of pulmonary and critical care medicine in China‐Japan Friendship Hospital. All cases underwent TBLC in a hybrid cone beam CT (CBCT) operation room with a single general anesthesia. The definitive diagnosis was made by a multidisciplinary team that involved clinicians, radiologists and pathologists. This study analyzed the biopsy sample surface areas, main complications and the consistency between TBLC pathology and multidisciplinary discussion (MDD) diagnosis for DIP.

**Results:**

An average of 3.1 ± 1.1 specimens were obtained per patient. The mean surface area of the specimen was 23.7 ± 6.1 mm^2^. None of the cases had pneumothorax or massive hemorrhage. Ten cases (83.3%) had no or mild bleeding and two cases (16.7%) had moderate bleeding. All cases had the typical pathologic characteristics of DIP, which was highly consistent with the diagnosis of MDD.

**Conclusion:**

TBLC can obtain sufficient samples for the pathological diagnosis of DIP, which has high security and accuracy in experienced specialist centers.

## 1 INTRODUCTION

Desquamative interstitial pneumonia (DIP) is a rare interstitial lung disease (ILD), which is first described by Liebow in 1965.^1^ Almost all patients of DIP are current or previous smokers. The common clinical manifestations are dry cough and progressive dyspnea. The primary feature is the presence of a diffuse and bilateral ground‐glass opacifications (GGO) with the lower lung lobes and peripheral distribution on high‐resolution computed tomography (HRCT). Other findings on HRCT may also be irregular linear, reticular opacities and small round changes. Cysts honeycomb and nodule appearance are not common.^2^, ^3^, ^4^ The bronchoalveolar lavage fluid shows numerous macrophages with pigment granules. Histopathology plays an important role in the diagnosis of DIP. The predominant histological feature is accumulation of brown pigmented macrophages in the alveolar space on different levels. These macrophages reveal eosinophilic red‐stained cytoplasm. Alveolar septal thickening is common. Although there is mild to moderate interstitial inflammation, the alveolar structure remains intact. Interstitial fibrosis is rarely seen.^5^, ^6^ Sufficient tissue specimens are the basis of accurate pathological diagnosis.

The surgical lung biopsy (SLB) is the recommend approach for obtaining lung tissue to identify histopathologic pattern for idiopathic interstitial pneumonias (IIPs) including DIP.^7^ Owing to invasiveness and high expense, SLB is difficult to be widely applied in clinical practice.^8^, ^9^ Transbronchial lung cryobiopsy (TBLC) is a new technique to obtain specimens and has been used for ILD diagnosis in recent years.^10^, ^11^, ^12^ Reports suggested that TBLC could offer almost equivalent contribution to the diagnosis of IIPs compared with SLB within the context of MDD.^11^ The pathological changes of DIP are relatively uniform. The lesions of DIP are mainly distributed in peripheral and lower lung lobes, which is conducive to TBLC to obtain tissue specimens.^2^ Dias reported five patients with clinical and radiological findings compatible with DIP underwent TBLC as part of an ILD diagnostic approach. TBLC confirmed the pathologic diagnosis of DIP in all five patients.^12^ The above content suggested that TBLC might be an alternative approach to achieve tissue samples for DIP diagnosis. In this article, we retrospectively evaluated the accuracy and safety of TBLC for DIP diagnosis in our clinical practice.

## 2 METHODS

### 2.1 Patients selection

A retrospective review was performed from January 2019 to December 2020 at the Department of Pulmonary and Critical Care Medicine of China‐Japan Friendship Hospital.162 patients underwent TBLC as a diagnostic approach of ILD. All patients should meet the following eligibility criteria: ≥18 and ≤75 years of age; the partial pressure of arterial oxygen >60 mmHg (1 mmHg = 0.133 kPa) while breathing room air, and forced vital capacity (FVC) >50% predicted, and diffusing capacity of the lung for CO (DLCO) >35% predicted. Key exclusion criteria were uncorrectable or excessive bleeding risk, pulmonary hypertension, and severe cardiopulmonary insufficiency. Twelve patients with confirmed DIP by MDD were included in this study (Table 1).

**TABLE 1:** Clinical data and HRCT characteristics of the selected patients

Case No.	Age	Sex	Smoking (py)	HRCT characteristics	FVC(/L)	FEV1/FVC (%)	DLCO‐SB (%)
1	58	M	40	Wide‐ranging GGO and emphysema in upper lobe traction bronchiectasis in bilateral middle and lower lobes	4.5	64	53.9
2	47	M	60	Wide‐ranging GGO and emphysema in upper lobe	4.4	86	65
3	56	M	30	Wide‐ranging GGO traction bronchiectasis in bilateral lower lobes	3.9	79	65
4	27	M	10	Multiple patchy GGO in bilateral lower lobes	4.8	82	64.9
5	55	M	40	Wide‐ranging GGO and emphysema in the upper lobe traction bronchiectasis in bilateral lower lobes	4.1	96	63.8
6	56	M	30–60	Wide‐ranging GGO traction bronchiectasis in bilateral lower lobes	1.3	90	52
7	75	M	60	Wide‐ranging GGO in bilateral lung lobes	3.3	56	105
8	64	M	44	Wide‐ranging GGO in bilateral lung lobes	3.45	75	81
9	38	M	44	Wide‐ranging GGO in bilateral lung lobes	2.68	80.1	51.4
10	40	M	15	Wide‐ranging GGO and subpleural nodules	2.89	30.5	37.5
11	71	M	10	Wide‐ranging GGO in bilateral lung lobes	4.08	72.13	78.7
12	60	M	60	Wide‐ranging GGO and scattered nodules	3.5	72.5	70

Abbreviations: DLCO, diffusing capacity of the lung for CO; FVC, forced vital capacity; GGO, ground‐glass opacification; HRCT, high‐resolution computed tomography; TBLC, transbronchial lung cryobiopsy.

Approval was obtained from the Medical Ethics Committee of China‐Japan Friendship Hospital. All patients gave written informed consent to enrollment in the study.

### 2.2 TBLC

**FIGURE 1:**
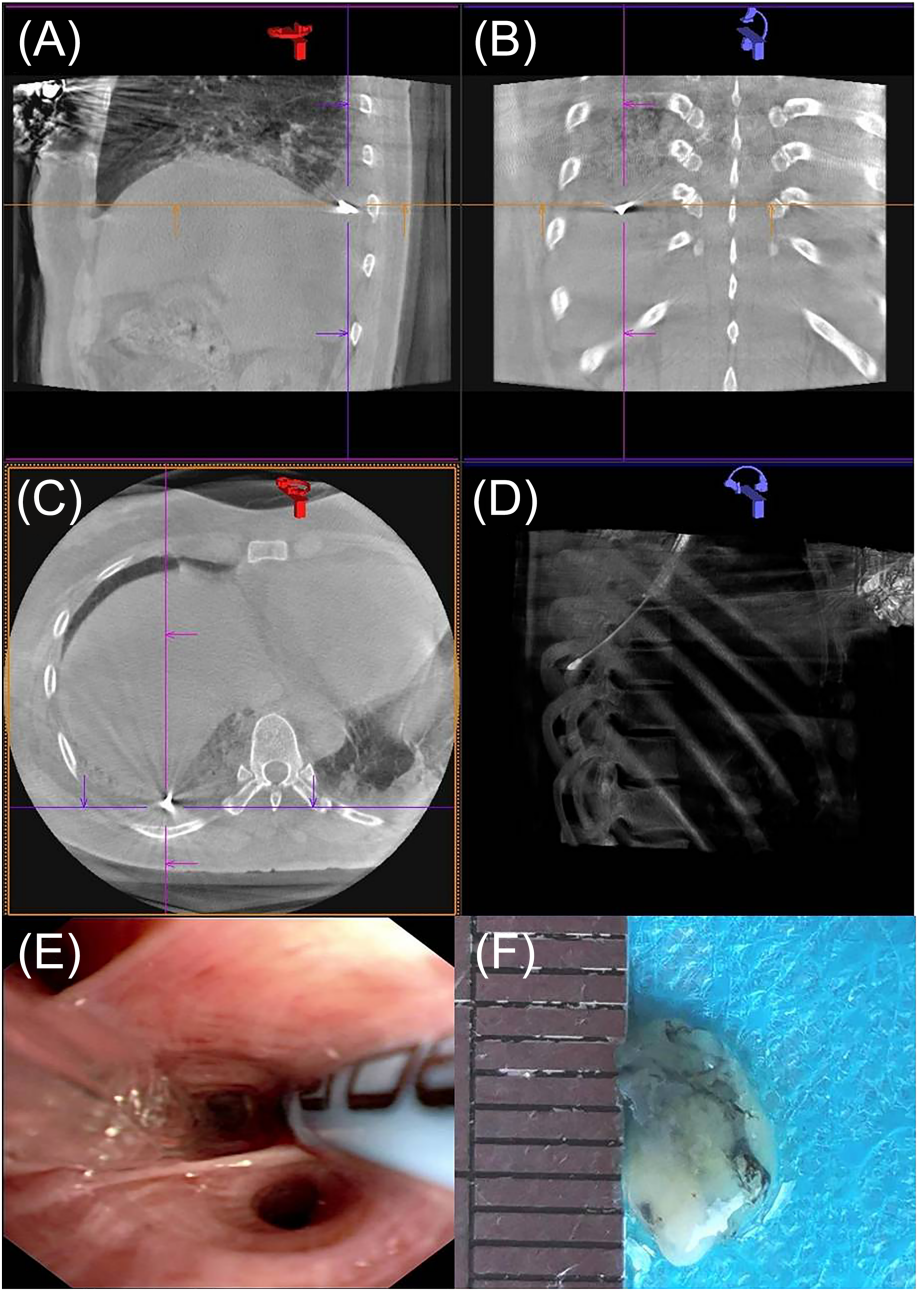
(A–D) Three‐dimensional computed tomography (CT) images were acquired to accurately assess the distance from probe‐to‐pleura; (E–F) The prophylactic balloon was placed near the target position under the guidance of the conducting wire and a specimen was obtained per patient by transbronchial lung cryobiopsy (TBLC)

The procedure was performed under deep sedation with intravenous propofol and remifentanil in a hybrid CBCT operation room. Under general anesthesia, patients were intubated with a rigid bronchoscopy. Through the bronchoscopy working channel, cryoprobe (ERBE, Solingen, Germany) was advanced into the target bronchial segment which was selected by the bronchoscopist in view of HRCT scanning. The cryoprobe should be retracted by 1–2 cm, if it encountered resistance. CBCT imaging (Artis Zee III ceiling, Siemens AG, Germany) was activated. Three‐dimensional computed tomography (CT) images were acquired to accurately assess the distance from probe‐to‐pleura. The position of the cryoprobe could be adjusted to ensure that the probe was 1 cm away from the pleura. The prophylactic balloon was placed near the target position under the guidance of the conducting wire in the meantime. Once the location was determined, the cryoprobe was performed (6–8 s freeze time for 1.9‐mm cryoprobe or 4–6 s for 2.4‐mm cryoprobe), using carbon dioxide as the cryogen. As a regular, the balloon would be activated after each sample. Cold physiological saline, thrombin, or adrenaline may be sprayed through the bronchoscope, if necessary. After the specimen was removed, CBCT would be applied again to determine whether there was pneumothorax. Before the endotracheal tube was removed, the bronchoscope was re‐entered the airway to determine whether there was no active bleeding. The tissue specimens were obtained in at least two different sites (either different segments in the same lobe or different lobes).^13^ The frozen biopsy specimen was first thawed in saline at room temperature and then transferred to formalin for fixation (Figure 1).

Electrocardiogram (ECG), blood pressure, and oxygen saturation were monitored throughout the whole process.

### 2.3 Pathologic assessment

The conclusions of pathologic specimen were interpreted by two pulmonary pathologists respectively, who were blinded to know the patient's clinical information and imaging characteristics. If they did not agree on the pathological judgments of some pathologic specimens, they would record a final diagnostic impression after reviewing the biopsy together again.

### 2.4 The standard of bleeding

No bleeding was defined as no active bleeding and presented only with the presence of traces.^14^

Mild bleeding was defined as negative pressure suction could automatically stop bleeding.

Moderate bleeding was defined as local injection of frozen saline, epinephrine, thrombin, or balloon occlusion required for hemostasis.

Severe bleeding was defined as the hemorrhage that could not be controlled under endoscopy, resulting in incomplete stability of hemodynamics and respiratory function, or admission to a critical care unit.

## 3 RESULTS

### 3.1 The specimen

TBLC was performed in posterior basal segment and outer basal segment of lower lobe predominantly. An average of 3.1 ± 1.1 specimens were obtained per patient. The mean surface area of the specimen was 23.7 ± 6.1 mm.^2^

### 3.2 Complication

None of the cases had pneumothorax or severe hemorrhage. Ten cases (83.3%) had no or mild bleeding and two cases (16.7%) had moderate bleeding. No active hemorrhage was observed after hemostasis under bronchoscope. There were no other postoperative complications occurred during the 30‐day follow‐up.

### 3.3 Histopathology

**FIGURE 2:**
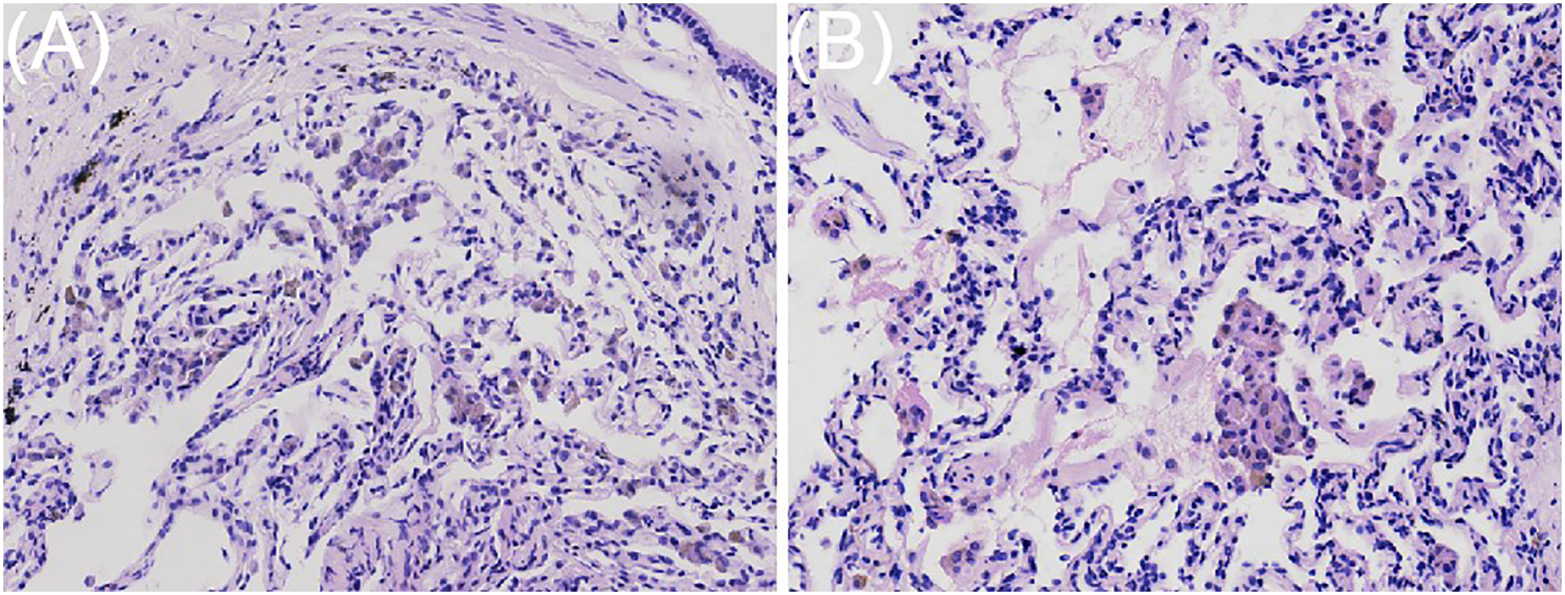
(A–B) The alveolar space was accumulated of brown pigmented macrophages at different levels, which contained eosinophilic cytoplasm. (H&E stain; magnification, ×100)

Under a low‐power microscope, the alveolar structure of the sample tissues was intact, and there was not significantly alveolar collapse and nuclear crush. Under a high‐power microscope, the alveolar space was accumulated of brown pigmented macrophages at different levels, which contained eosinophilic cytoplasm. Alveolar septal thickening is caused by lymphocytes, plasma cells, and eosinophils. The alveolar structure remained intact in spite of the inflammatory changes. Interstitial fibrosis was observed in some cases (Table 2, Figure 2).

**TABLE 2:** Specimen data and pathologic characteristics of the selected patients

Case No.	Number and location of TBLC samples	Size of TBLC specimen (mm^2^, mean value)	The degree of bleeding	Histopathology
1	4	24.3	No bleeding	Alveolar septa widened, fibrous tissue hyperplasia, lymphocyte infiltration, a large number of macrophages phagocytic tobacco particles in alveolar cavities, focal fibroblast proliferation
2	3	25.3	No bleeding	Alveolar septa widened, fibrous tissue hyperplasia, a small amount of chronic inflammatory cells and eosinophils infiltration, brown pigmented macrophages filled in alveolar cavities
3	4	23	No bleeding	Extensive peribronchiolar metaplasia, interstitial fibrosis, brown pigmented macrophages aggregation in some alveolar cavities
4	5	16.2	No bleeding	Focal alveolar septa slightly widened, interstitial fibrous tissue hyperplasia with lymphocytes infiltration, brown pigmented macrophages scattered and aggregated in alveolar cavities
5	3	29	Moderate bleeding	Alveolar septa slightly widened, interstitial fibrosis with infiltration of lymphocytes and plasma cells mildly, brown pigmented macrophages scattered and aggregated in alveolar cavities
6	4	31.25	Mild bleeding	Alveolar septa widened, a few lymphocytes and eosinophils infiltrated, a large number of brown pigmented macrophages in some alveolar cavities
7	2	26	Moderate bleeding	Focal widened alveolar septa, brown pigmented macrophages in most of the alveolar cavities
8	2	35	Mild bleeding	Alveolar septa slightly widened, a few chronic inflammatory cells infiltrated, brown pigmented macrophages in alveolar cavities
9	2	20	Mild bleeding	Focal alveolar septal thickened, chronic inflammatory cells infiltration, fibrous tissue proliferation, brown pigmented macrophages in alveolar cavities
10	4	16	Mild bleeding	Alveolar septa widened, interstitial fibrosis accompanied by unequal lymphocytes infiltration, a large number of brown pigmented macrophages in alveolar cavities
11	2	22.5	No bleeding	Focal widened alveolar septa, lymphocytes and plasma cells infiltration, brown pigmented macrophages in alveolar cavities
12	2	16	No bleeding	Peribronchiolar metaplasia, alveolar septa widened, fibroblast proliferation, brown pigmented macrophages in alveolar cavities

### 3.4 Multidisciplinary discussion

A multidisciplinary team was comprised of clinicians, radiologists and pathologists with ILD expertise. They evaluated each patient in combination with clinical characteristics, HRCT, and the histopathology. Among the 162 patients that had undergone TBLC in this study, five of them were preliminarily diagnosed as DIP in combination with clinical and radiological features. In the MDD, correlation of clinical, radiological, and histopathologic findings yielded a definite DIP diagnosis in three cases. In addition, nine cases confirmed with DIP by MDD were diagnosed respectively smoking‐related interstitial pneumonia (two cases), nonspecific interstitial pneumonia (four cases) and chronic hypersensitivity pneumonitis (three cases) before TBLC.

## 4 DISCUSSION

DIP is a type of the smoking‐related IIPs. Carrington identified that the alveolar space was accumulated of pigmented macrophages conventionally known as smoker's macrophages, which contain eosinophilic cytoplasm and distinct light brown pigmentation.^15^

Histopathology plays an important role in the diagnosis of ILD. Although international guidelines suggest SLB for patients when the combination of the clinical characteristics and the imaging patterns of HRCT cannot provide a definite diagnosis,^7^ clinicians still hesitant to adopt SLB on the cause of significant complications and appreciable expense in practice. Poor cardiopulmonary reserve, advancing age, and comorbid disease often render patients with ILD unsuitable candidates for SLB.^8^, ^9^ Of patients with unclassified interstitial pneumonia, 52% did not receive SLB due to fear of high risk. Thus, diagnostic uncertainty has an adverse impact on treatment.^16^

TBLC is a novel technique for sampling lung tissue for ILD diagnosis.^17^ TBLC can obtain tissue specimens approximately 10–30 mm^2^, which is enough to observe the structure of primary lung lobules.^10^ In this study, the average tissue size obtained by TBLC was 23.7 ± 6.1 mm^2^ consistent with the literature report. The more specimens we obtain, the higher ILDs diagnosis rate may be. SLB can obtain larger lung tissue, which leads to a higher diagnostic yield of SLB than that of TBLC (approximately 95% for SLB VS 80% for TBLC).^13^ The current gold standard for the diagnosis of ILD is determined by a multidisciplinary group of professional clinicians, radiologists, and pathologists. Recent reports suggested that TBLC may offer equivalent contribution to the diagnosis of ILD compared with SLB within the context of MDD. Troy suggested that the consistence of histopathological pattern and MDD diagnosis were respectively 70.8% and 76.9% between TBLC and SLB. With definite or high diagnostic confidence, the concordant diagnoses were as high as 95% for TBLC at MDD.^11^ Harari et al. indicated that the proportion of diagnoses between TBLC and SLB was 88% and 90%, respectively.^18^ Compared with SLB, the relatively small biopsy tissue is the main limitation of TBLC in ILDs pathological diagnosis. But for particular ILDs with a uniform temporal appearance, the size of qualified TBLC specimens can theoretically meet the histopathologic diagnostic need. The most common finding of DIP is ground‐glass opacity on HRCT, which has a peripheral subpleural and basal predominance.^2^ The characteristics of distribution and histopathological uniformity of DIP are suitable for TBLC to acquire tissue specimens. In 2020, Barata et al. describe their experience with TBLC in diagnostic work‐up of patients with smoking‐related ILDs. Forty‐five patients were included, and the most frequent histopathologic pattern found was DIP (33 patients).^19^ There are no clinical trial data available to date to evaluate diagnostic yield of TBLC on DIP. But we found that TBLC and SLB show similar diagnostic efficiency for DIP/RB‐ILD in well‐designed studies.^11^, ^20^ In our study, 60% of the five patients who were preliminarily diagnosed as DIP before TBLC were ultimately diagnosed with definite DIP in the MDD. Another nine patients confirmed with DIP were initially diagnosed with other types of IIP in the absence of pathology. These presented that TBLC could be considered a reliable diagnostic tool in this particular scenario in the context of MDD.

TBLC has good security with a lower percentage of complications compared with SLB, especially in mortality.^20^ Pneumothorax and bleeding was the most common complication after TBLC. In published meta‐analyses of TBLC, the reported range of substantial bleeding is wide, at 0.3%–26.6% of cases. In this study, we noticed none of the patients had severe hemorrhage. Moderate bleeding events was observed in two patients (16.7%) that was immediately treated by endoscopic measures. The absence of severe bleeding may be related to the initiation of balloon to inflate immediately after the biopsy specimen is removed from the airway. Studies have shown that the degree of lung fibrosis is positively correlated with the risk of bleeding after TBLC. Our patients had no obvious fibrosis on HRCT, which may be related to less bleeding.

A systematic review of the literature found that pneumothorax occurred in about 20% of cases after TBLC. Peripheral HRCT fibrosis score was positively correlated with the risk of pneumothorax. Harari found that the incidence of pneumothorax was 10.9% in their study.^18^ In our review, no pneumothorax occurred intraoperatively, and no pneumothorax or death occurred during the 30‐day follow‐up. This may be related to the small number of cases we enrolled. More importantly, experienced operators determined the precise localization under guidance of CBCT before starting the cryoprobe in our study. Zhou indicated that the incidence of pneumothorax was only 1.9% through taking the same technique in a relatively large sample.^21^ With less complications, the patients had a shorter hospital stay and cost less, which made TBLC increasingly popular in clinical practice as a potential alternative to SLB.

**FIGURE 3:**
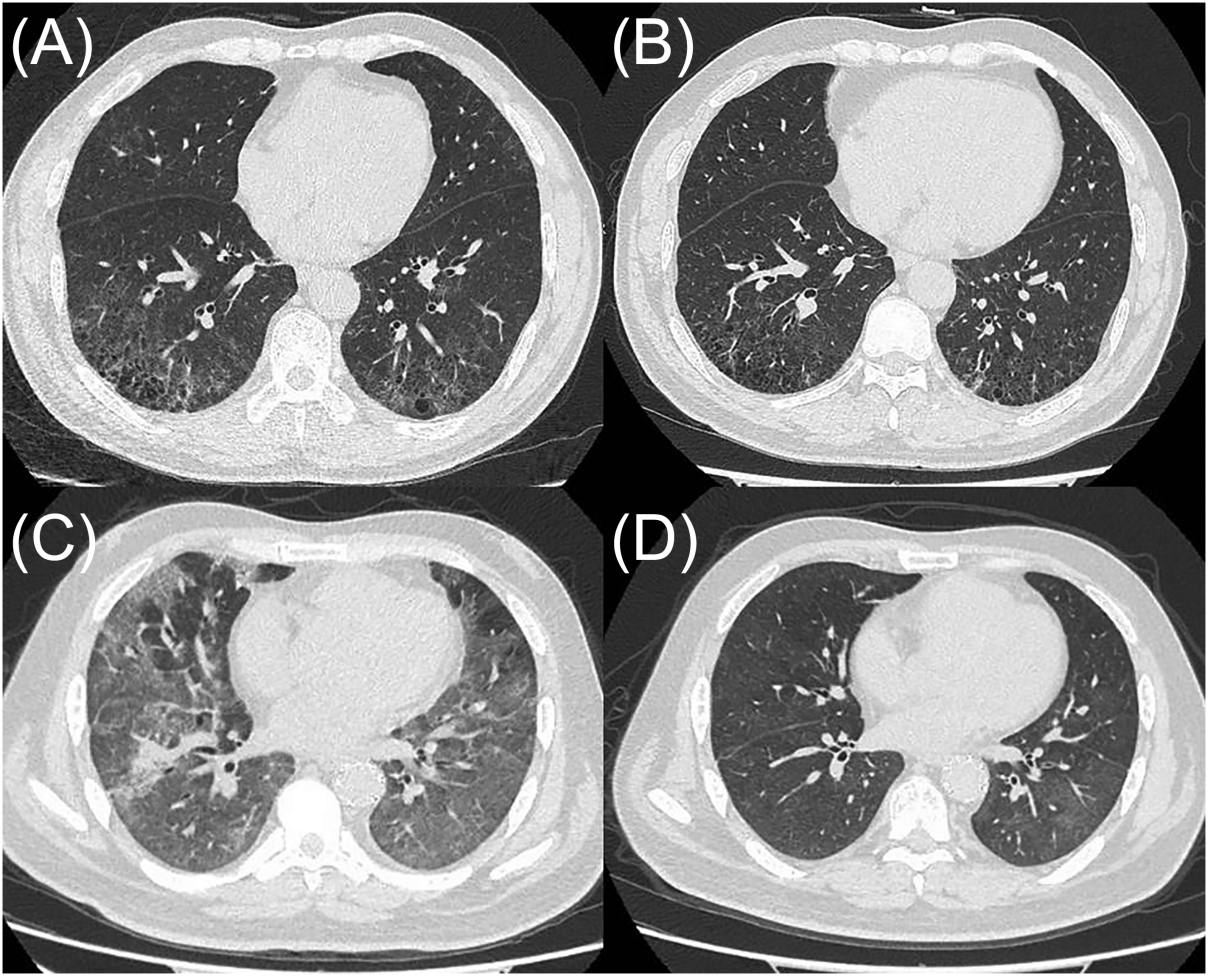
High‐resolution computed tomography (CT) of the chest: (A, C) Images of the two patients showed different degrees of diffuse ground glass opacity and traction bronchiectasis before treatment (B, D) the ground glass opacities decrease and traction bronchiectasis regressed obviously after treatment

After MDD evaluation, the definitive diagnosis for all cases was DIP. Once diagnosed, the cessation of smoking or occupational exposure is currently the primary treatment. DIP patients may be treated with long‐term glucocorticoids and or immune suppressants or lung transplants.^22^ In our study, almost all patients received a certain amount of glucocorticoid. In consideration of fibrotic in alveolar septa, two patients received anti‐fibrosis treatment.^23^, ^24^ The symptoms mentioned gradually improved and the GGO at the lower lobes regressed to some extent at the 3‐month follow‐up (Figure 3).

## 5 CONCLUSION

Our study demonstrates that TBLC with less invasion can obtain sufficient samples for the pathological diagnosis of DIP, which also has high security and accuracy. This is a summary of our experience in clinical work, which needs to be further confirmed by prospective design and clinical studies with SLB as control.

## CONFLICT OF INTEREST

The authors have no conflicts of interest to disclose.

## AUTHOR CONTRIBUTIONS

Yan‐hong Ren designed the study and revised the manuscript. Guo‐wu Zhou, Ling Zhao, and Min Liu collected the data. Jun‐ge Zhao analyzed the data and wrote the paper. Hua‐ping Dai contributed to the review of the manuscript.

## ETHICS STATEMENT

Approval was obtained from the Medical Ethics Committee of China‐Japan Friendship Hospital. All patients gave written informed consent to enrollment in the study.

## Data Availability

The data that support the findings of this study are available from the corresponding author upon reasonable request.
